# Amorphous Ropinirole-Loaded Mucoadhesive Buccal Film: A Potential Patient-Friendly Tool to Improve Drug Pharmacokinetic Profile and Effectiveness

**DOI:** 10.3390/jpm10040242

**Published:** 2020-11-25

**Authors:** Giulia Di Prima, Giuseppina Campisi, Viviana De Caro

**Affiliations:** 1 Dipartimento di Scienze e Tecnologie Biologiche Chimiche e Farmaceutiche (STEBICEF), Università degli Studi di Palermo, 90123 Palermo, Italy; giulia.diprima@unipa.it; 2 Dipartimento di Discipline Chirurgiche, Oncologiche e Stomatologiche, Università degli Studi di Palermo, 90127 Palermo, Italy; giuseppina.campisi@unipa.it

**Keywords:** orocomucosal films, buccal administration, ropinirole, parkinson’s disease, therapy optimization, ex vivo permeation, mucoadhesion, Eudragit^®^ L100, dissolution kinetics, Peppas-Salhin model

## Abstract

Nowadays the therapeutic strategies to manage Parkinson’s Disease are merely symptomatic and consist of administering L-DOPA and/or dopamine receptor agonists. Among these, Ropinirole (ROP) is a widely orally-administered molecule, although it is extensively susceptible to hepatic metabolism. Since literature reports the buccal mucosa as a potentially useful route to ROP administration, the development of novel, effective, and comfortable oromucosal formulations should prove desirable in order to both enhance the therapeutic efficacy of the drug and allow a personalized therapeutic strategy able to meet the patient’s needs. The results of the proposed ROP film as a new dosage form show that it is flexible; uniform; and characterized by suitable surface pH; good mucoadhesiveness; low swelling degree; and fast, complete drug release. Moreover, after ex vivo evaluation on a film having an area of 0.282 cm^2^ and dose of 2.29 mg, the results of drug flux through the buccal mucosa are closely comparable to the amount of ROP that reaches the bloodstream at the steady-state condition after ROP-PR 4 mg oral administration, calculated according to the literature (0.237 mg/cm^2^·h^−1^ vs. 0.243 mg/h, respectively). Moreover, drug flux and ROP dose could be accurately modulated time-by-time depending on the patient’s need, by varying the administered disk area. In addition, the proposed ROP film displays no lag time, producing an immediate drug input in the bloodstream, which could result in a prompt therapeutic response. These findings make ROP film a potentially comfortable and patient-friendly formulation, and a promising candidate for further clinical trials.

## 1. Introduction

Oromucosal films (mucoadhesive buccal films or orodispersible films) are relatively new kinds of formulations among the variety of pharmaceutical dosage forms for personalized medicine. These types of medications should be employed for the treatment of certain local or systemic disorders, such as oral inflammatory pathologies or central nervous system diseases, respectively, and could improve patients’ compliance due to a lower dosing frequency. Moreover, some crucial features such as the possibility of taking them with no or just a little water makes oromucosal films suitable drug delivery systems for patient populations with special needs, e.g., with swallowing difficulties or in late-stage Parkinson’s Disease (PD) [1,2,3].

In particular, mucoadhesive buccal films are placed into the mouth and attach to the buccal mucosa. To achieve systemic effects, the drug released from the formulations have to be absorbed via the mucosa, bypassing the gastrointestinal tract. However, adequate mucoadhesion of the formulation is a crucial precondition for buccal absorption [3]. Recently, there have been a number of advances in formulation development to improve the retention and absorption of drugs in order to overcome the limitation of conventional buccal dosage forms (liquid e.g., drops and sprays or semi-solids e.g., gels). Indeed, the latter are commonly affected by physiological factors such as salivation and swallowing, thus reducing the adhesion of the formulation on the mucosa and leading to unpredictable drug absorption [4]. At this point, matrix mucoadhesive buccal films are usually patient-friendly as they are soft and deformable, as well as characterized by thinness. They generally consist of an ingredient combination such as polymer, plasticizer, sweetener, and other necessary additives and could incorporate drug compounds of different natures, such as highly water-soluble or poorly water-soluble low molecular weight drugs, nutraceuticals, or herbal plant extracts [1,5,6].

The administration of these formulations benefits from the great advantages of the buccal route, such as its non-invasive nature and ease of administration. Additionally, the buccal route has been shown to be an effective alternative to the traditional oral one, especially in the following cases: (i) fast onset of action is required (drugs can be rapidly and directly absorbed into systemic circulation via venous drainage to the superior vena cava); (ii) patients have swallowing difficulties as in late-stage PD; (iii) patients have severe gastric motility deficiencies so that medicines rest for hours in the stomach leading to no-on or delayed-on effects, as in late-stage PD; (iv) administration of drugs susceptible to pH and enzymatic degradation, such as occurs in the gastrointestinal tract; (v) administration of drugs that undergo high first-pass hepatic metabolism [4,7,8].

In this view, Ropinirole (ROP) is a suitable candidate to be administered via the buccal route. Ropinirole (4-[2-(dipropylamino)ethyl]-1,3-dihydro-2H-indol2-one) is a non-ergoline dopamine agonist which has a high affinity for and stimulates the post-synaptic dopamine receptors D_2_ in the central and peripheral nervous systems. In current clinical practice, ROP is employed in monotherapy or together with L-DOPA to treat PD. PD is a progressive neurodegenerative pathology that involves a gradual loss of dopaminergic neurons, particularly in the substantia nigra pars compacta, leading to both cognitive and motor decline. Nowadays, there is no cure for PD, and current therapy is symptomatic, largely based on ‘replacing’ the deficit in dopamine transmission that is associated with the disease motor pathology. Replacement therapy comprises the administration of L-DOPA (a dopamine precursor) able to reach the Central Nervous System and be transformed into dopamine, thus releasing it from spared presynaptic terminals, and/or dopamine receptor agonists that directly activate dopamine receptors. The direct action on postsynaptic dopamine receptors bypasses the requirement for functioning presynapses and proves crucial in late-stage PD [9,10]. Among the dopamine receptor agonists, ROP is approved for use as monotherapy in the treatment of early-stage PD and as an adjunct to L-DOPA therapy when more advanced-stage disease occurs. Currently, ROP is administrated orally and two kind of formulations are available: Ropinirole immediate release (ROP-IM) and Ropinirole prolonged release (ROP-PR). In particular, by administering ROP-IM, ROP is rapidly and almost completely absorbed (T_max_ 1–2 h after dosing), however, its bioavailability is nearly 50% due to extensive first-pass-effect metabolism. Considering that circulating metabolites do not contribute to ROP pharmacological effect and that the ROP half-time is about 6 h, ROP-IM must be taken three times daily. This results in fluctuations of plasma drug concentration throughout the day, poor control of PD symptoms, and low patient compliance. To overcome these limitations, ROP-PR formulations have been developed. The once-daily dosing of ROP-PR provides continuous delivery of ROP over the 24 h, resulting in a smooth plasma concentration–time profile, increasing tolerability profile, and improvements in motor function, as well as in the ability to perform the activities of daily living. Moreover, compared with therapies that are administered two or three times daily, ROP-PR formulations increase patients’ compliance. However, it has been reported that this type of preparation allows the achievement of steady-state conditions after 48 h [11,12,13,14,15,16,17,18].

Since there is evidence that the ROP is capable of passively permeating the oral mucosa [19], over the last years many efforts have been made in order to propose mucoadhesive buccal formulations [20,21,22]. Furthermore, it is known that the polymeric matrix systems are able to enhance the solubility and absorption of the actives by their transformation in amorphous form [23]. As a consequence, the design and development of an effective oromucosal matrix system in the form of a film should result in a novel, well-accepted therapeutic option. 

In particular, the aim of the present work was to design, prepare, and characterize a mucoadhesive polymeric buccal film in order to meet the need of continuous individualization of therapeutic dosages to manage PD. Indeed, considering the therapeutic needs of PD patients and the susceptibility of ROP to hepatic first-pass effect after oral administration, the proposed formulation should exhibit a lower dose, a faster absorption (ideally with no lag time), and a rapid achievement of the steady-state conditions in order to minimize plasma concentration fluctuations respective to the oral formulation. Moreover, by a strong and prolonged adhesion of a flexible film to the retromandibular trigone of buccal mucosa, where salivary turnover is slow, it is possible to maximize drug absorption minimizing drug loss in the gastrointestinal tract due to swallowing. Finally, by changing the size of the film, the amount per hour (flux) of ROP that enters the systemic circulation could be adjusted time-by-time, modulating it on the patient’s symptoms.

In order to evaluate the potential effectiveness of the proposed mucoadhesive buccal film our findings were compared with pharmacokinetics parameters reported after ROP-PR administration. The results reported here suggest that the developed ROP film could result in an optimization of treatment outcomes in terms of efficacy, safety, and quality of life.

## 2. Materials and Methods

### 2.1. Materials

Ropinirole hydrochloride (ROP) was kindly supplied by Teva (Petach Tikva, Israel). Eudragit^®^ L100 was kindly supplied by Rofarma (Milan, Italy). Trehalose dihydrate was purchased from Hayashibara Shoij (Hayashibara Shoij Inc., Okayama, Japan). Polyvinylpyrrolidone (PVP) K-90, triacetin, triethanolamine (TEA), and agar were purchased from Sigma-Aldrich Chemie (Stenheim, Germany). Ascorbic acid and propylene glycol were purchased from Farmalabor (Canosa di Puglia, Italy). Phosphate buffer saline (PBS) Ca^2+^ and Mg^2+^ free (simulated plasma pH 7.4) were prepared by dissolving KH_2_PO_4_ (0.144 g/L), anhydrous Na_2_HPO_4_ (0.795 g/L), and NaCl (9.0 g/L) in distilled water. The saline isotonic solution was prepared by dissolving NaCl (0.9% w/v) in distilled water. The simulated salivary fluid (pH 6.8) was prepared by dissolving NaCl (0.126 g/L), KCl (0.964 g/L), KSCN 0.189 g/L), KH_2_PO_4_ (0.655 g/L), urea (0.200 g/L), Na_2_SO_4_∙10H_2_O (0.732 g/L), NH_4_Cl (0.178 g/L), CaCl_2_·2H_2_O (0.228 g/L), and NaHCO_3_ (0.631 g/L) in distilled water [23]. The components used for the preparation of buffers were purchased from VWR International (Milan, Italy). Chemicals were of analytical grade and solvents were of HPLC grade (HiPerSolv Chromanorm). All chemicals and solvents were used without any further purification. Porcine mucosae specimens were kindly supplied by the Municipal Slaughterhouse of Villabate (Palermo, Italy).

### 2.2. Methods

#### 2.2.1. Preparation of ROP-Loaded Matrix Film

ROP-loaded matrix films were prepared by the solvent casting method. Firstly, two separated solutions (A and B) were prepared and then mixed. Solution A was prepared by dissolving propylene glycol (80 mg), PVP K-90 (16 mg), and TEA (32 mg) in distilled water (5 mL). After complete dissolution, trehalose (80 mg), NaOH 1 M (3 mL), and Eudragit^®^ L100 (480 mg) were added under continuous stirring until a clear, homogeneous, and viscous solution was obtained. Solution B was prepared by dissolving ROP (80 mg) and ascorbic acid (6 mg) in distilled water (5 mL). Finally, the clear solution B was gently added to solution A under continuous stirring and then triacetin (50 mg) was added. A clear, light-yellow solution with an increased viscosity was obtained. The mixture was then carefully and slowly put into a silicone mold (area 8 cm^2^), in order to avoid bubbles formation, and then placed into the oven (StabiliTherm, Thermo Scientific, Waltham, MA, USA) for 48 h at 30 °C. Afterwards, the obtained dry matrix film was left at room temperature for 24 h. From each matrix film, small disks (diameter: 0.6 cm; area: 0.283 cm^2^) were used for further characterizations and studies were obtained using a biopsy punch. The obtained small disks were packed into polyethylene bags, heat-sealed, and stored at room temperature in a glass container to maintain the integrity and elasticity of the films.

#### 2.2.2. Uniformity of ROP-Loaded Matrix Films (Drug Content, Weight, and Thickness)

The prepared matrix films were characterized in terms of uniformity of drug content, thickness, and weight variation.

Drug content was evaluated by dissolving each small disk sample in a 100 mL flask filled with artificial plasma pH 7.4, sonicated (Branson B1200, Branson Ultrasonic Corporation, Danbury, CT, USA) until a clear solution was obtained, and brought to volume with the same fresh solvent. The amount of ROP contained in each disk was measured spectrophotometrically (UV/VIS Shimadzu model 1700 instrument, Japan) at λ = 250.0 nm. ROP standards were used as references to construct the calibration curve (concentration range: 0.005–0.100 mg/mL; E_1%_ = 0.304; y = 30.81143, x − 0.01213; R^2^ = 0.9999). Data are reported as amount of drug (mg) into 100 mg of drug-loaded matrix film (drug loading percentage—DL%) ±SE. The weight variation test (Mettler AE240 balance, Mettler-Toledo AG, Greinfensee, Switzerland) was performed in accordance to Italian Pharmacopoeia (FUI XII ed.). Data are reported as the weight of the total matrix film prepared ± SE (in order to verify the reproducibility of the preparation procedure) as well as small disks weight values ± SE (in order to verify the homogeneity of each matrix film). Matrix film thickness was measured in five different points of each film by a digital micrometer (VWR International, Milano, Italy) having a measuring range from 0 to 25 mm (sensibility 0.01 mm). Data are reported as thickness ± SE.

#### 2.2.3. Folding Endurance

To evaluate the flexibility of buccal films, folding endurance was assessed by repeatedly folding a specified region of each film at the same point until it broke or was folded to 300 times (end point) without breaking. The number of folding allowed for each film was reported as folding endurance value [24]. 

#### 2.2.4. Evaluation of Surface pH

Preliminarily, an agar plate was prepared by dissolving 2% (w/v) agar in warmed simulated salivary fluid (pH 6.8) under stirring, and then pouring the solution into a Petri dish until it gelled at room temperature. Randomly selected film disks were put on the surface of agar plate, covered with 0.5 mL of simulated salivary fluid and left to swell for 2 h. Then, surface pH was measured using a pH meter (HI 2211 pH/ORP Meter, Hanna Instrument, Woonsocket, RI, USA) by placing pH probe in closely contact with the wetted film surface. The experiment was performed in duplicate for each film (n = 8). 

#### 2.2.5. Swelling Test and Radial Erosion

The swelling behavior as well as the radial erosion of the ROP film were evaluated by placing a disk on a glass support positioned on a graph paper. The experiment was started by placing 200 μL of simulated salivary fluid (pH 6.8, 37 °C) on the disk and adding 100 μL of saliva every 5 min for the first 15 min, and then every 15 min. At every time interval a photograph was taken to evaluate any change in the disk’s morphology.

#### 2.2.6. Differential Scanning Calorimetry (DSC)

DSC analysis was performed on the film disks as well as on their individual components by using a Perkin Elmer Jade calorimeter. Samples (of approximately 8 mg) were tested in the temperature range of 30–250 °C at a heating rate of 10 °C/min, under a continuous nitrogen flow.

#### 2.2.7. In Vitro Dissolution Studies and Drug-Release Kinetics Evaluation

Film dissolution in simulated salivary fluid (pH 6.8) was assessed using the flow-through system previously described [25]. Briefly, the system consists of a beaker containing simulated saliva (100 mL) thermostated at 37 °C, from which the liquid is forced into a Plexiglass release chamber by a peristaltic pump (Bio-Rad Econo Pump, Hercules, CA, USA). The flow rate of saliva was maintained constant at 0.5 mL/min. In the chamber was allocated a film disk of 1.1 cm diameter sandwiched between two cellulose acetate membranes to avoid fragments of films clogging tubes. In the chamber, the salivary layer wetting the sides of the disk was about 0.2 mm thick. The temperature was maintained at 37 ± 0.1 °C by submerging the chamber in a thermostatic bath. Aliquots (1 mL) of solution coming out from the release chamber were collected every 2 min and the drug amount was then quantified by UV-VIS detection, using the appropriate blank and calibration curve (concentration range of standard solutions: 0.005–0.025 mg/mL; y = 30.92 x − 4.93·10^−3^; R^2^ = 0.9996). Results are reported as mean of six experiments and graphically elaborated as percent amount of drug released ± SE versus time (min). Each experiment was carried out until the complete dissolution of the disk occurred (data resulted in accordance with those previously observed during the disintegration studies and the radial erosion experiments). The amount of drug released matched the original drug content of the disk.

The release data were elaborated by Origin 8.5 software (OriginLab Corporation, Northampton, MA, USA) and fitted to the semi-empirical equation usually applied to evaluate drug release from delivery systems [26]. Fittings were validated by using R^2^ and χ^2^ where a *p*-value of less than 0.05 was considered statistically significant.

#### 2.2.8. Ex Vivo Mucoadhesive Strength Measurement

The ex vivo mucoadhesive strength evaluation was performed by the modified two-armed physical balance method [27]. Porcine buccal mucosa excised from just-slaughtered pigs was used as model tissue and handled without any pre-treatment. A tissue portion of the inner part of the cheek was glued by cyanoacrylate resin (Super Attak Loctite^®^, Henkel Italia Srl, Milan, Italy) on a glass Petri dish support and maintained at 37 ± 1 °C for the whole experiment. The film disk was fixed using a bi-adhesive to the lower side of a rubber stopper hanging from the balance arm. Before starting the measurements, the mucosal tissue was wetted with 50 µL of simulated salivary fluid and then the film was placed on the tissues so that it was just touching the mucosal surface. A light force was applied for 20 s with a fingertip. The measurements were carried out considering different contact times after the film application on the mucosa (10, 15, 20, 30, 40, and 50 min). The grams required to detach the disk from the mucosal surface provided the measurement of mucoadhesive strength, as following:

Force of adhesion (N) = (g − 9.81)/1000

Afterwards, the mucoadhesive properties were expressed as detachment force, namely the force required to separate 1 m^2^ of film from the mucosa, calculated as:

Detachment force (N/m^2^) = Force of adhesion (N)/Surface area (m^2^)

The experiment was performed in triplicate [28,29]. 

#### 2.2.9. Ex Vivo Permeation Studies

The permeation studies were performed using vertical jacketed Franz-type diffusion cells (Permeagear, flat flange joint, 9-mm orifice diameter, 15-mL acceptor volume, SES GmbH—Analysesysteme, Bechenheim, Germany), used as a two-compartment open model. As membrane models, mucosal specimens consisted of tissue removed from the inner cheek (buccal area) of freshly slaughtered eight-month-old domestic pigs intended for human consumption were used. After sampling, all specimens were immediately placed in PBS (pH 7.4), transferred to the laboratory in a refrigerated transport box within 1 h, surgically treated to remove the excess of adipose and connective tissues within 2 h of the animal sacrifice and then stored at −20 °C for periods up to one week. Before the experiments, specimens were equilibrated at room temperature and dipped for approximately 1 min in a pre-warmed (60 °C) saline solution; the connective tissue was then carefully peeled off from the mucosa (slides 250 ± 25 mm thick) to obtain the heat-separated epithelium along with the intact basal lamina [19]. The thickness was measured using a digital micrometer. Then, specimens were equilibrated in PBS for about 3 h at room temperature, the PBS replaced with fresh PBS every 15 min to remove biological matter, which could interfere with drug analyses. Appropriate sections of tissue (12 mm disks diameter) were mounted in vertical Franz-type diffusion cells and equilibrated for 1 h at 37 ± 0.1 °C, adding PBS in both the donor and the acceptor compartments. This step was followed by the removal of PBS from the donor compartment, replacement with a film disk, and a soaking using 0.4 mL of simulated salivary fluid pH 6.8. At regular time intervals (60 min), samples (0.5 mL) were withdrawn from the acceptor compartment and immediately replaced with fresh PBS in order to maintain sink conditions. Each experiment was carried out at 37 ± 0.1 °C for 6 h. Results were reported as means ± SE of six experiments. The amount of ROP permeated through the porcine buccal mucosa was determined by UV-VIS analysis as described above.

#### 2.2.10. Data Analysis

Data were expressed as mean ± SE. All differences were statistically evaluated by the Student’s t test with the minimum levels of significance with *p* < 0.05.

## 3. Results and Discussion

### 3.1. Design, Development, and Optimization of ROP-Loaded Film

The aim of the present study was to homogenously embed ROP into a bioerodible matrix film in order to obtain a new buccal dosage form able to improve ROP absorption, avoid the first-pass effect and thus increase its bioavailability.

Generally, a buccal matrix film is based on an appropriate ingredient combination, such as polymer, plasticizer, drug, and other necessary additives. All components have to be accurately dosed in order to design an effective and patient-friendly formulation which should be: (i) soft, comfortable, and flexible, as well as characterized by thinness, in order to not interfere with normal functions such as drinking, talking, etc.; (ii) fast dissolving, in order to quickly provide the required drug dose; (iii) strongly mucoadhesive, in order to avoid drug loss; (iv) able to enhance drug absorption while providing unidirectional and controlled drug release toward the buccal mucosa [30].

To ensure the desired characteristics, the choice of the film-forming agent is crucial. In particular, Eudragit^®^ L-100 was chosen as film-forming polymer. Eudragit^®^ L-100 (Methacrylic Acid Copolymer Type A USP/NF, Methacrylic Acid—Methyl Methacrylate Copolymer 1:1 Ph. Eur.) is a biocompatible, mucoadhesive, anionic, and water-soluble polymer, frequently used to prepare oral-controlled release formulations. Its water solubility is closely influenced by the environmental pH due to the presence of the acidic residues, which rends it insoluble in an acidic environment but soluble at neutral or alkaline pH (solubility increases above pH = 6) [31,32,33,34]. Moreover, Eudragit^®^ L-100 has the interestingly ability to reduce the precipitation rate of poorly water-soluble molecules through a steric hindrance mechanism, due to the high molecular weight as well as the presence of long side chains [35]. This feature could prove very useful because of the poor water solubility of ROP in the indissociated form; which, however, is the readily absorbable one through the buccal mucosa.

Considering Eudragit^®^ L-100’s behavior in aqueous media, the use of a base (sodium hydroxide) is required in order to neutralize the acidic residues and allow it first to dissolve polymers and then to form a viscous gel [36]. Furthermore, after salification the environment remains weakly alkaline, thus shifting the balance of ROP dissociation towards the indissociated form. To further guarantee the maintenance of a weakly alkaline environment, triethanolamine (TEA) has also been added to the formulation. Additionally, the introduction of polyvinylpyrrolidone (PVP) is crucial in order to obtain an effective adhesive drug delivery system. Thanks to its ability to form additional bonds with the mucins and its high biological and chemical inertia, PVP is able to ensure a prolonged an intimate contact with the buccal mucosa [37,38]. Moreover, the addition of plasticizing agents is needed to obtain a flexible and soft film that could prove comfortable and adaptable to the site of application. Trehalose, propylene glycol, and triacetin were then chosen due to their ability to retain water and simultaneously promote absorption through the mucosa [39]. Finally, ascorbic acid was added to the formulation as a preservative agent due to its antioxidant and antimicrobial properties [40].

After design and preparation, uniformity studies (in terms of weight, thickness, and drug loading) were performed to assess the accuracy of dose in the film and the reproducibility of the preparation method.

The considered parameters are reported in Table 1 and highlight high film reproducibility and homogeneity, thus confirming the soundness of the preparation technique (data are in accordance with F.U. XII ed.—mass and drug content uniformity assay for oral solid dosage forms).

### 3.2. ROP-Loaded Film Characterization

Ideally, a buccal film should be characterized by the following features: (i) it should be soft and flexible, with an appropriate surface pH and a low swelling degree in order to be comfortable and patient-friendly; (ii) it should contain the drug in the amorphous and thus readily soluble/absorbable form while avoiding the formation of drug crystals; (iii) it should exhibit an appropriate and reproducible dissolution and drug-release rate, which have to be rapid but simultaneously must also give time to the formulation to firmly adhere to the mucosa in order to minimize drug loss in the oral cavity; (iv) it should be adequately mucoadhesive in order to ensure a stable and prolonged contact with the surface of application/absorption. All these crucial parameters could be evaluated by the following tests respectively: (i) folding endurance, surface pH, and radial erosion evaluation; (ii) differential scanning calorimetry (DSC) analysis; (iii) dissolution test simulating the in vivo salivary turnover; (iv) mucoadhesive strength evaluation.

#### 3.2.1. Comfort of ROP Film

**Figure 1:**
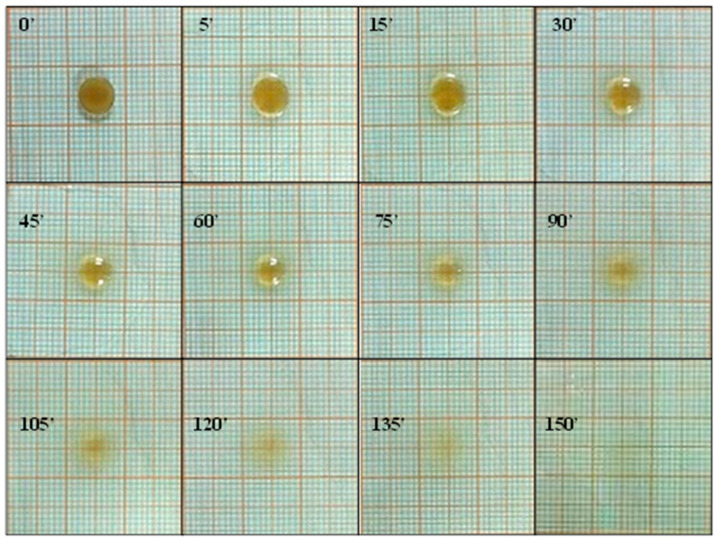
Swelling degree and radial erosion studies of the ROP film: photographs of ROP film at the selected time intervals.

The flexibility of the ROP film was assessed, as it did not show any cracks even after folding more than 300 times. In accordance with the literature, these data were considered satisfactory to reveal good formulation plasticity properties [41]. The evaluation of the surface pH is crucial to establish the compatibility of the proposed formulation with the buccal mucosa. Actually, in order to guarantee film suitability, the surface pH must be compatible with the physiological pH of the application site: an excessively acid or alkaline environment could cause irritation or adverse phenomena. The average surface pH for the ROP film was 6.8 ± 0.25, thus also film compatibility with the application site was successfully assessed. Finally, swelling degree and radial erosion tests were performed to help us to understand the morphological changes affecting the formulation after administration, due to the contact with the oral cavity fluids. The performed studies are “dynamic” tests as, at time intervals, aliquots of fresh simulated salivary buffer were added in order to promote swelling and/or erosion and made these phenomena evaluable. Results (Figure 1) highlight a starting slight film swelling due to the initial absorption of the simulated salivary buffer, following a slow rate film erosion, until complete dissolution of the matrix system. 

The collected data confirm the potential comfort of ROP films after administration as they are soft and deformable, show a surface pH compatible with the buccal mucosa, and exert a very low swelling degree, thus being promising in terms of patient compliance and tolerability.

#### 3.2.2. DSC Analysis

**Figure 2:**
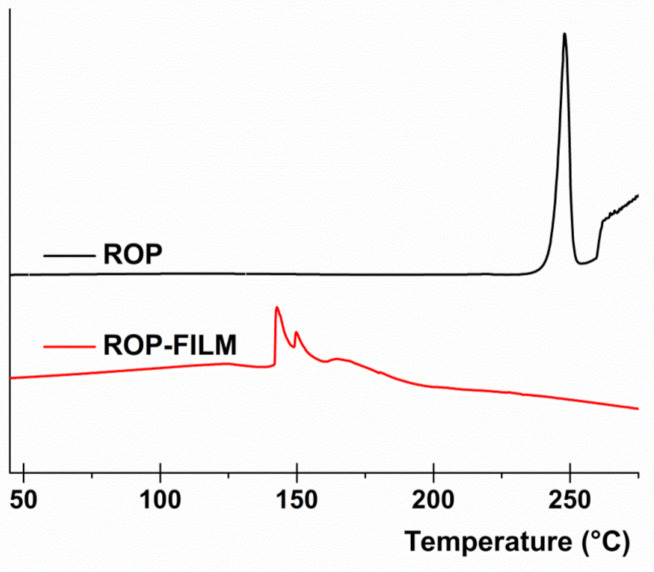
Differential Scanning Calorimetry (DSC) analysis: thermograms of pure crystalline ROP (black line) and ROP film (red line).

In order to evaluate the physical state of the ROP when it is embedded into the matrix film, Differential Scanning Calorimetry (DSC) analysis was conducted. In particular, Figure 2 reports the DSC thermogram of pure crystalline state ROP compared with the thermogram of the obtained ROP film. As observable, the pure crystalline ROP thermogram exhibit a net and well-defined endothermic peak at 249 °C corresponding to the drug melting point, as expected. In contrast, the obtained thermogram registered for the ROP film did not show any comparable endothermic peak, thus suggesting that ROP is embedded into the matrix film as amorphous. These data are quite satisfactory as this way ROP solubility and consequently absorption should be enhanced.

#### 3.2.3. Dissolution Studies

As the first step leading to drug absorption through the buccal mucosa involves drug dissolution in the biological fluids, knowledge of the drug-release profile in simulated salivary fluid is crucial to provide a solid rationale for therapeutic effectiveness. Usually, drug-release studies are performed according to the official pharmacopoeias. However, these methods (characterized by large volumes as well as the maintenance, or sink or pseudo-sink conditions) do not efficiently simulate the in vivo conditions prevailing in the oral cavity environment. Indeed, this case is characterized by small amounts of liquid and non-sink conditions. For these reasons, in the present study, drug-release studies were performed using a flow-cell system able to simulate the buccal conditions and, in particular, the salivary turnover in the oral environment [25].

**Figure 3:**
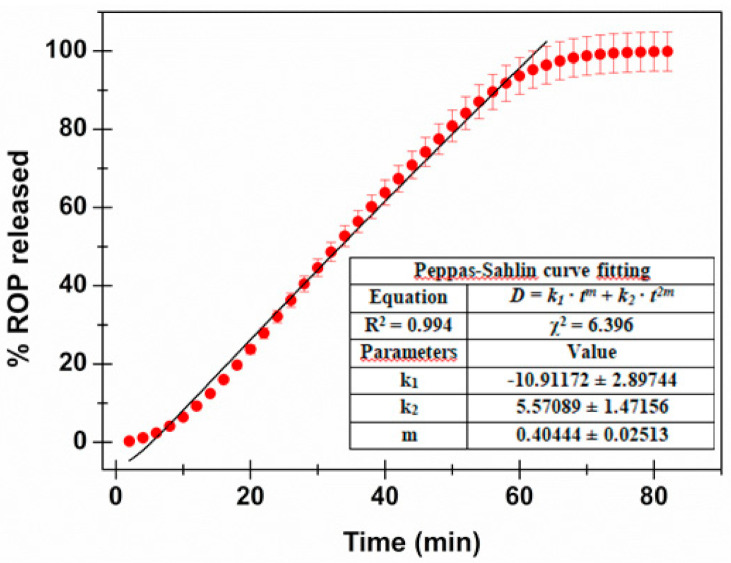
ROP release profile: percentage of drug released as a function of incubation time. The Peppas–Salhin fitting is highlighted together with the calculated fitting parameters.

The obtained drug-release behavior is reported in Figure 3. As observable, the drug-discharge trend tends to a plateau after about 64 min with 96.4% of drug released), until the total amount of loaded ROP is completely released (99.9%) after about 80 min.

In order to better understand the mechanism of the in vitro drug release, data were fitted with different mathematical models. In particular, the zero order, first order, Higuchi, Hopfenberg, Korsmeyer–Peppas (power law) and Peppas–Salhin models, usually used in dissolution analysis, were curve-fitted to the experimental data [26,42,43]. Table 2 reports the fitting parameters, the square of correlation coefficients (R^2^), and chi-square (χ^2^) obtained for each of the mentioned mathematical models.

As expected, the Higuchi model (Equation (3)) did not fit well (R^2^ = 0.758). This is due to the assumption, when considering this model, that the matrix is inert and thus swelling and/or erosion is negligible. However, as already evaluated by swelling studies, the formulation proposed here absorbs water, swells, and then undergoes erosion. Consequently, the Hopfenberg model (Equation (4)), usually employed to explain the drug-release behavior from erodible polymers, was considered. This mechanism gave better fitting results (R^2^ = 0.976); however, considering the knowledge about the proposed matrix composition, together with data obtained by the swelling studies, further mathematical models were explored. In particular, the Korsmeyer–Peppas (power law—Equation (5)) and the Peppas–Salhin (Equation (6)) models were considered. Power law is a comprehensive semi-empirical equation to describe drug release from polymeric systems when the release mechanism is not known or when more than one type of phenomenon of drug release is involved. Depending on the value of *n* that better adjusts to the release profile, it is possible to establish whereas drug discharge is Fickian or non-Fickian [42]. When *n >* 1 (obtained *n* value: 1.177 ± 0.037), the Super Case II model, consisting in an extreme form of transport, occurs. In this case, during the absorption process, tension and breaking of the polymer chains happens and this is in accordance to the previously obtained experimental data. The adherence to this model is confirmed by the high R^2^ value obtained (0.987). In any case, the best fitting results were those obtained with the Peppas–Salhin model (Equation (6)). This starts from the power law model but allows us to approximately calculate the contribution of two different mechanisms (diffusion and relaxation/erosion) in an anomalous drug-release process. The equation related to the Peppas–Salhin model could be divided as follow: the first term represents the Fickian diffusional contribution, F, whereas the second term represents the Case II relaxational/erosional contribution, R. When the erosion mechanism is negligible, the *m* values are equal to the *n* of the power law. For the ROP-loaded films proposed here, as erosion significantly contributes to drug discharge, *m* ≠ *n*. In particular the *m* value was extrapolated by the fitting analysis (until the plateau is reached: 64 min—96.4% of drug released) as calculated following the method described by Peppas and Salhin in 1989 (by the aspect ratio and the related graph and then fitting until the first 60% of drug release). However, in both cases, the values obtained are superimposable: 0.404 ± 0.025 vs. 0.415 respectively. When considering the *m* value of 0.415, the curve fitting results are quite similar for the power law and the Peppas–Salhin models (R^2^ 0.987 and 0.988, respectively). Definitely, the best-fitting results were obtained by applying the Peppas–Salhin model and curve-fitting data until the plateau (R^2^ = 0.994), thus confirming the accordance with the model until the exhaustion of the formulation (Figure 3). In order to better understand the diffusion and relaxation/erosion contribution to drug-release behavior, the F and R parameters were calculated as follows [44]:

$$F=\frac{1}{1+\;\frac{k_{2}}{k_{1}}t^{m}}$$

$$\frac{R}{F}=\frac{k_{2}\cdot t^{m}}{k_{1}}$$

**Figure 4:**
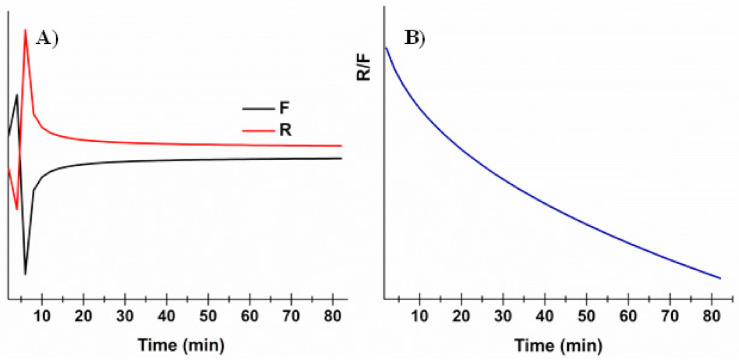
Evaluation of the Fickian diffusional contribution (F) and the Case II relaxation/erosional contribution (R) in the Peppas–Salhin model: (**A**) variation of the Fickian diffusional contribution (F) and Case II relaxation/erosional contribution (R) as a function of time; (**B**) variation of the R/F ration as a function of time.

The obtained data are reported in Figure 4 and highlight that drug release is initially due to the Fickian contribution, while over time the relaxation/erosional contribution becomes predominant. This is in accordance with results obtained by the swelling studies: (i) during the starting slight film swelling due to the initial absorption, presumably diffusion through the matrix is the main mechanism, and then (ii) while film erosion occurs, the relaxation contribution increases. This results in the decrease of the R/F ratio over time (Figure 4, panel B).

#### 3.2.4. Mucoadhesive Strength

The oral cavity (with a 0.1–0.7-mm-thick mucus layer) represents an important route of administration for mucoadhesive dosage forms. For buccal applications, the knowledge of the dissolution profile and drug-release rate is not sufficient to confirm the appropriateness of the dosage form because, depending on the dosage form, some drug loss can occur due to swallowing of saliva and/or the retention time on the application site could be relatively low due to the continuous salivary washout [45]. As a consequence, the strong adhesion of the film on the mucosal surface is another fundamental requirement in order to (i) create high drug concentration directly on the absorption site while minimizing drug loss into the oral cavity; (ii) ensure the permanence of the formulation on the mucosal surface avoiding its detachment and deglutition that would otherwise cause patients discomfort as well as drug loss into the gastrointestinal tract. The force of adhesion expresses the mass (g) necessary to detach the film from the mucosal surface and could be converted into detachment force by being normalized per unit area, as reported in the Materials and Methods section. As mucoadhesion is a multi-stage process, it is fundamental to consider the initial contact time as a crucial parameter [46]. 

Consequently, Table 3 reports the calculated force of adhesion and corresponding detachment force for progressive contact times (from 10 to 50 min). As expected, a trend is observable as the force of the adhesion/detachment force initially increases exponentially until tending to a plateau for prolonged contact times (40 min). The choice to end the experiment at 50 min is due to the achievement of the plateau together with data obtained from dissolution studies (see above). Moreover, it has to be considered that this type of experiment, even for prolonged contact times, is quite static and does not require the addition of simulated salivary fluid. Thus, the area to be considered to calculate the detachment force is that of the film disk (no addition of fluid results in no disk dissolution).

**Figure 5:**
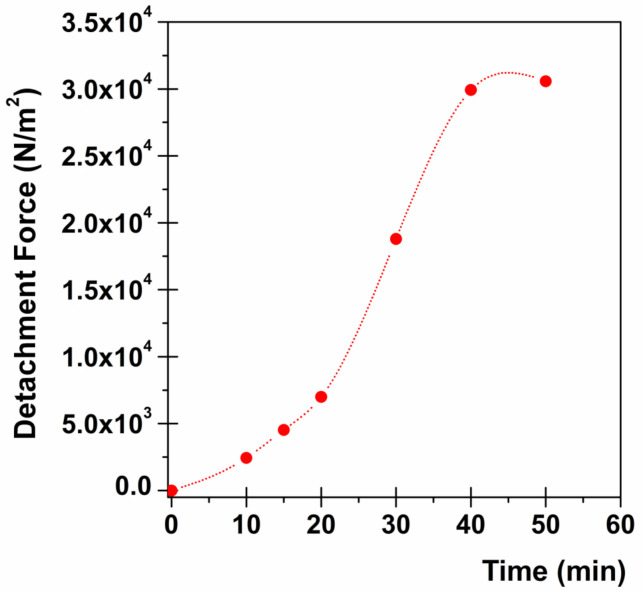
Mucoadhesion studies: relationship between contact time and detachment force (the standard error obtained results so small that the error bars are not visible in the graph).

To encourage a better and immediate interpretation, data from Table 3 are graphically presented in Figure 5, in order to highlight the mucoadhesive behavior of ROP film.

### 3.3. Ex Vivo Studies and Pharmacokinetic Considerations

To evaluate the potential effectiveness of ROP film, ex vivo permeation studies were performed employing porcine buccal mucosa, as it is considered the most comparable to human tissue in terms of lipid content, thickness, composition, and morphology (e.g., non-keratinized epithelium) [47].

Actually, as the aptitude of ROP to cross the buccal mucosa is already known in the literature, the aim of this work was to embed ROP in an appropriate solid formulation in order to benefit from the advantages of a mucoadhesive matrix buccal film [19].

**Figure 6:**
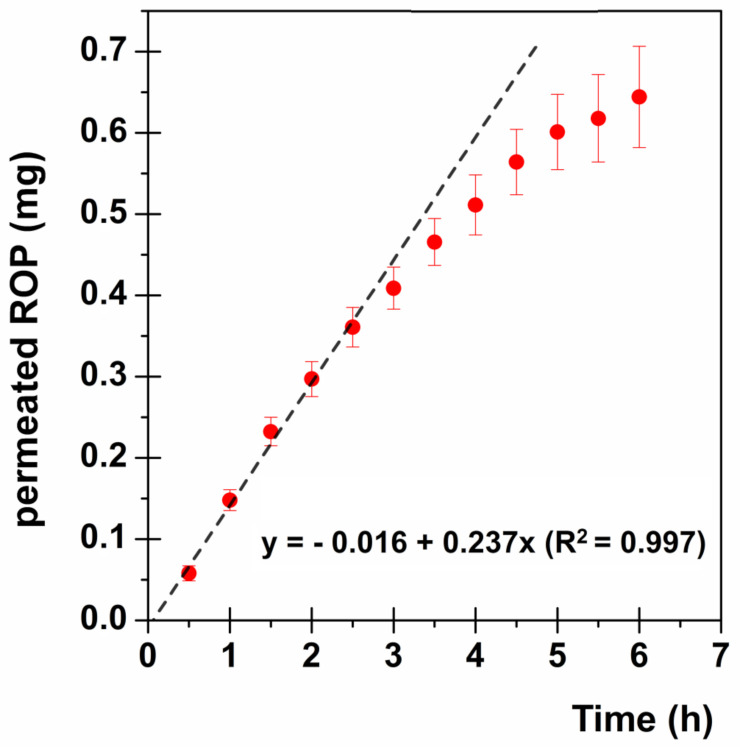
ROP permeation profile through the buccal mucosa: mg/cm^2^ of permeated drug as a function of time (●). The linear fitting at the steady state is highlighted (--).

The ROP permeation profile expressed as mg/cm^2^ of drug permeated as a function of time is reported in Figure 6.

In order to evaluate the effectiveness of the proposed formulation as a therapeutic option, first of all drug flux (J_s_) through the buccal mucosa was calculated as follows:

$$J_{s}=\frac{Q}{At}(mg/cm^{2}\cdot h)$$

where Q is the amount of drug permeated in the time interval t and A is the active area available for absorption.

At the steady state J_s_ is equal to the slope of the straight line obtained. In particular, as the graph in Figure 6 shows, the ROP permeation profile is characterized by no lag time and a maintenance of the steady state until about 2.5 h, followed by the slowing down of the permeation, probably due to the experimental conditions, which do not maintain the sink conditions anymore. Consequently, for further evaluations the steady-state flux [0.237 mg/(cm^2^·h)] will be considered.

To correct and complete evaluate the obtained results, as well as to compare the formulation proposed here with those currently employed (ROP-PR 4 mg or 8 mg), it is necessary to take into account the ROP pharmacokinetic parameters:

(a) [Cp]_ss_ (Plasma Concentration at the steady state): = 1 ng/mL/mg;
(b) t_1/2_ = 6 h (as a consequence K_e_ = 0.693/t_1/2_ = 0.1155 h^−1^);
(c) V_D_ = 7.5 L/Kg (as a consequence V_D_ for a 70 Kg average adult results 525 L) [11,12,19,48,49].

At the steady state, the rate of drug absorption is equal to the rate of drug elimination and thus, considering the V_D_ parameter, it is possible to calculate the ROP amount per hour needed to maintain the plasma concentration constant and effectiveness. In particular:

Drug Elimination Rate (D_E_) = [Cp]_ss_ ·K_e_

Drug Absorption Rate (D_A_) = D_E_·V_D_

Considering ROP-PR 4 mg, the [Cp]_ss_ normalization to the dose is 4 ng/mL. Therefore, multiplying [Cp]_ss_ by the known elimination constant K_e_, the obtained D_E_ is equal to 0.462 ng/(mL·h). Consequently, it is possible to calculate the amount of ROP that must reach the systemic circulation to maintain plasma concentration constant and comparable to ROP-PR 4 mg, by considering the V_D_ for an average 70 kg adult. The obtained D_A_ results 0.243 mg/h. Similarly, when considering ROP-PR 8 mg, it is possible to calculate the required D_A_ value, which results in 0.485 mg/h.

The proposed buccal formulation shows a J_s_ comparable to the D_A_ value calculated to maintain plasma concentration at the steady state as ROP-PR 4 mg [0.237 mg/(cm^2^·h) vs. 0.243 mg/h]. However some crucial considerations have to be pointed out: i) for the ex vivo experiments, a small disk having a dose of 2.29 mg of ROP was employed; ii) even if the starting administered dose is lower (2.29 mg < 4 mg of ROP-PR) the ROP absorption rate is quite similar; iii) as the administered disk is very small (0.282 cm^2^) it is possible to increase the ROP flux through the mucosa by applying a larger film disk which contains a higher ROP amount; iv) drug absorption profile through the buccal mucosa (Figure 6) shows no lag time producing an immediate drug input bloodstream flux comparable to the needed drug influx to maintain the steady-state concentration. This could result in a prompt therapeutic response.

Other relevant considerations are closely related to the buccal route of administration. In particular, drug flux (J_s_) depends on the area of absorption and this parameter was considered according to the experimental setup (0.636 cm^2^). However, the in vivo conditions are quite different, as the proposed buccal film should be placed at the level of the retromandibular trigone where one face of the film adheres to the buccal surface, while the other is in close contact with the gingival mucosa. This could result in enhanced drug absorption and high drug flux. Subsequent clinical trials will be needed to evaluate the behavior of the film.

## 4. Conclusions

To briefly summarize, the present work reports the design, development, and characterization of a novel mucoadhesive buccal film loaded with Ropinirole and potentially useful as an innovative strategy to be employed in the treatment of Parkinson’s Disease in order to meet the need of continuous individualization of novel therapeutic approaches. The here-proposed buccal films are composed of Eudragit^®^ L100, plasticizers, preservative agent, and additionally mucoadhesive polymer, and proves able to incorporate ROP in its amorphous form. The solvent casting method was chosen to prepare the ROP film and led to a uniform preparation in terms of weight, thickness, and drug content. Moreover, further analysis assessed the potential comfort of the preparation as the buccal film proved soft and flexible, low-swellable, strongly mucoadhesive, and able to release the total drug amount embedded. Finally, the ex vivo permeation studies gave consistent results in terms of the pharmacological potentiality of the proposed ROP film as the administration of a very small film disk (0.282 cm^2^—2.29 mg ROP) proved comparable to the conventional ROP-PR 4 mg oral dosage form in terms of drug absorption rate at the steady state and even better, as, in this case, no lag time is observable. Indeed, this way it is possible to administer a lower dose to achieve the same Cp because by the buccal administration method, the hepatic first-pass effect is avoided. Furthermore, Cp fluctuations should be avoided, as by administering the buccal film, drug absorption occurs immediately and quickly. Additionally, as the administered disk is very small (0.282 cm^2^) it is possible to increase ROP flux through the mucosa by applying a larger film disk containing, as consequence, a higher starting ROP amount. These data suggest that the developed ROP film could result in an optimization of treatment outcome in terms of efficacy, safety, and quality of life. In brief, the proposed formulation should become a potential patient-friendly tool as: (i) it could be comfortable as suggested by its flexibility (low discomfort), low swelling degree (low discomfort), and strong mucoadhesiveness (low risk of detachment and/or loss), which could result in high patient compliance; (ii) it could guarantee a fast and good therapeutic efficacy as ROP is immediately absorbed, the hepatic first-pass effect is avoided and drug flux is adequate; (iii) it could meet patients’ special needs in terms of both feasibility of administration in patients with swallowing difficulties and/or severe gastric motility deficiencies due to PD progression and easy modulation of drug dose and flux by varying time-by-time the dimension of the film to be administered on the patient’s symptoms. These findings make ROP film a promising candidate for further clinical trials.

## Figures and Tables

**Table 1:** Characteristics of Ropinirole- (ROP-)loaded film in terms of uniformity (weight, thickness, and drug content ± SE).

Total film weight (8.04 cm^2^)	1.03 ± 0.04 g
Small disk film weight (0.282 cm^2^)	30.94 ± 0.50 mg
Thickness	793 ± 22 µm
Drug content	7.40 ± 0.13% (*w*/*w*) 2.29 ± 0.04 mg (per small disk)

**Table 2:** Mathematical models fitted to the experimental ROP release curve: calculated fitting parameters, the square of correlation coefficient (R^2^), and chi-square (χ^2^). Data were fitted until the plateau is reached (64 min—96.4% of drug released), except in the Peppas–Salhin *** case (60% of ROP released).

Equation Number	Fitting Equations	Calculated Parameter(s)	R^2^	χ^2^
(1)	**Zero Order** ***D* = *k*·*t***	*k*: 1.533 ± 0.035	0.962	29.989
(2)	**First Order** ***D* = 100·(1 − *e*^−*k t*^)**	*k*: −0.024 ± 0.002	0.865	145.406
(3)	**Higuchi** ***D* = *k*·*t*^0.5^**	*k*: 9.832 ± 0.497	0.758	260.997
(4)	Hopfenberg * ***D* = 100·[1 − (1 − *k*·*t*)*^n^*]**	*k*: 0.016 ± 2.4·10^−4^	0.976	25.419
(5)	**Korsmeyer-Peppas** **(Power Law)** ***D* = *k*·*t^n^***	*k*: 0.782 ± 0.112 *n*: 1.177 ± 0.037	0.987	13.598
(6)	Peppas-Salhin ** ***D = k*_1_·*t^m^* + *k*_2_·*t*^2*m*^**	*k*_1_: −10.912 ± 2.897 *k*_2_: 5.571 ± 1.472 *m*: 0.404 ± 0.025	0.994	6.396
(6)	Peppas-Salhin *** ***D = k*_1_·*t^m^* + *k*_2_·*t*^2*m*^**	*k*_1_: −10.856 ± 0.792 *k*_2_: 5.264 ± 0.209 ***m*: 0.415**	0.988	4.677

* *n* = 1 because the studied formulation is a film, in accordance to the theoretical model; ** the *m* value was extrapolated by the fitting analysis; *** the *m* value was calculated after the determination of the aspect ratio (2a/l = diameter/thickness) by consulting Figure 1 reported by Peppas and Salhin (1989). As here reported, data were curve fitted until the first 60% of ROP was released.

**Table 3:** Force of adhesion and corresponding detachment force ± SE for progressive contact time.

Contact Time (min)	Force of Adhesion (N)	Detachment Force (N/m^2^)
10	0.069 ± 3.1·10^−4^	2435.11 ± 11.12
15	0.128 ± 5.8·10^−4^	4522.34 ± 20.65
20	0.196 ± 9.0·10^−4^	6989.36 ± 31.92
30	0.530 ± 2.4·10^−3^	18,785.11 ± 85.78
40	0.844 ± 3.9·10^−3^	29,917.02 ± 136.61
50	0.862 ± 3.9·10^−3^	30,581.56 ± 139.64
